# Molecular imaging of the brain–heart axis provides insights into cardiac dysfunction after cerebral ischemia

**DOI:** 10.1007/s00395-022-00961-4

**Published:** 2022-10-24

**Authors:** Nele Hermanns, Viola Wroblewski, Pablo Bascuñana, Bettina Wolf, Andras Polyak, Tobias L. Ross, Frank M. Bengel, James T. Thackeray

**Affiliations:** Department of Nuclear Medicine, Hannover Medical School, Carl Neuberg-Str. 1, 30625 Hannover, Germany

**Keywords:** Stroke, Positron emission tomography, Magnetic resonance imaging, Translocator protein, Heart failure

## Abstract

Ischemic stroke imparts elevated risk of heart failure though the underlying mechanisms remain poorly described. We aimed to characterize the influence of cerebral ischemic injury on cardiac function using multimodality molecular imaging to investigate brain and cardiac morphology and tissue inflammation in two mouse models of variable stroke severity. Transient middle cerebral artery occlusion (MCAo) generated extensive stroke damage (56.31 ± 40.39 mm^3^). Positron emission tomography imaging of inflammation targeting the mitochondrial translocator protein (TSPO) revealed localized neuroinflammation at 7 days after stroke compared to sham (3.8 ± 0.8 vs 2.6 ± 0.7 %ID/g max, *p* < 0.001). By contrast, parenchyma topical application of vasoconstrictor endothelin-1 did not generate significant stroke damage or neuroinflammatory cell activity. MCAo evoked a modest reduction in left ventricle ejection fraction at both 1 weeks and 3 weeks after stroke (LVEF at 3 weeks: 54.3 ± 5.7 vs 66.1 ± 3.5%, *p* < 0.001). This contractile impairment was paralleled by elevated cardiac TSPO PET signal compared to sham (8.6 ± 2.4 vs 5.8 ± 0.7%ID/g, *p* = 0.022), but was independent of leukocyte infiltration defined by flow cytometry. Stroke size correlated with severity of cardiac dysfunction (*r* = 0.590, *p* = 0.008). Statistical parametric mapping identified a direct association between neuroinflammation at 7 days in a cluster of voxels including the insular cortex and reduced ejection fraction (*ρ* = − 0.396, *p* = 0.027). Suppression of microglia led to lower TSPO signal at 7 days which correlated with spared late cardiac function after MCAo (*r* = − 0.759, *p* = 0.029). Regional neuroinflammation early after cerebral ischemia influences subsequent cardiac dysfunction. Total body TSPO PET enables monitoring of neuroinflammation, providing insights into brain–heart inter-organ communication and may guide therapeutic intervention to spare cardiac function post-stroke.

**Supplementary Information:**

The online version contains supplementary material available at 10.1007/s00395-022-00961-4.

## Introduction

Cerebral stroke is a major cause of global morbidity and mortality [35], but adverse consequences are not restricted to the brain. Specifically, increasing evidence supports detrimental influence of acute cerebral ischemia on cardiac function [8]. In addition to higher incidence of acute myocardial infarction (MI) in the first year after stroke [14], the risk of cardiac mortality over 2–6 years is double that of the healthy population [34]. While some risk is attributed to shared vascular risk factors of stroke and MI, concurrent development of heart failure suggests the presence of other common underlying pathogenetic mechanisms.

On one hand, cerebral stroke affects acute cardiac function, whereby neural damage in specific regions including the insular cortex directly affects heart rate and ventricular pressure [29]. However, stroke is also associated with inflammatory cell activation [32], including infiltration of circulating monocytes and macrophages to the core-surrounding penumbra in the days following injury. This amplified neuroinflammation increases the risk of secondary complications after stroke [9, 26], suggesting that inflammation may be another link between heart and brain after ischemic cerebral damage. Inter-organ communication within the heart–brain axis contributes to concurrent injury after focal ischemic damage, as evidenced after myocardial infarction with simultaneous neuroinflammation [5, 27, 40]. Similar crosstalk mechanisms may contribute to cardiac dysfunction after focal stroke, but can be difficult to assess serially using the conventional assays.

The mitochondrial 18kD-translocator protein (TSPO) is highly expressed by cerebral microglia, peripheral macrophages, and dysfunctioning myocytes [7]. It is a feasible target for whole-body non-invasive positron emission tomography (PET), using specific radioligands, which facilitate investigation of multi-organ networking after ischemic stroke [6, 40].

We hypothesized that cerebral stroke elicits neuroinflammation with concomitant systemic inflammation, and that the extent and location of stroke would influence cardiac outcome. We tested our hypothesis by serial TSPO-targeted PET in combination with multi-organ magnetic resonance (MR) imaging, and ex vivo validation by immunostaining and flow cytometry.

## Materials and methods

### Animals

All animal experiments were conducted in accordance with national research regulations and with the approval of the local state authority (Niedersächsiches Landesamt für Verbraucherschutz und Lebensmittelsicherheit (LAVES)). Male C57Bl/6 N mice (Charles River, *n* = 112) were housed in a temperature-controlled facility with a 14 h/10 h light/dark cycle and free access to standard laboratory diet and water.

### Study design

Stroke was induced by two surgical methods to generate a range of stroke severity and size: transient intraluminal occlusion of the middle cerebral artery (MCAo, *n* = 64) [11], or craniotomy with topical application of the vasoconstrictor endothelin-1 (ET-1) to the cortical parenchyma (*n* = 23) [43]. Animals were compared to age-matched sham surgeries as controls (MCAo sham, *n* = 18, topical vehicle replacing ET-1, *n* = 7). Selection of stroke model and related control surgery was determined randomly. All animals underwent serial multimodality imaging including TSPO PET imaging at acute (24 h), subacute (7 days), and chronic (21 days) timepoints after stroke. Brain and cardiac MR was performed early (2–4 days) and late (20-22d) after injury. To validate imaging signals, additional groups of animals were sacrificed at intermediate timepoints for ex vivo tissue workup. MCAo resulted in 25% mortality within the first 7 days of experiments, accounting for the drop-off of total animals across the timecourse. Otherwise, no animals were excluded from the study. Reconstructed images and histological sections were assigned an alphanumeric code and operators were blinded to the surgical condition of each subject for image analysis as far as possible. Representative images were selected based on alignment with quantitative median of the group, unless otherwise stated.

### Middle cerebral artery occlusion

MCAo surgery was performed as previously described [11]. Briefly, mice were anesthetized under isoflurane, and a midline incision was made between the manubrium sterni and the jaw to expose the left central carotid artery and the external carotid artery. Both were ligated to arrest blood flow and prevent blood leakage. Next, an arteriotomy was performed in the central carotid and a silicone-coated filament (7–0, Doccol Corporation, MA, USA) was introduced in the artery and advanced through the internal carotid artery (ICA) until it blocked the middle cerebral artery (MCA). A loose snare around the internal carotid was tightened to secure the filament. After 30 min, the territory was reperfused by steady withdrawal of the filament. An additional group of mice underwent a shorter duration of occlusion (20 min, *n* = 7) to generate a less extensive stroke. For sham surgeries, the filament was advanced to the MCA and immediately withdrawn. Upon removal of the filament, the internal carotid snare was retightened to prevent further bleeding from the arteriotomy and the incision was closed.

### Topical endothelin-1

Topical application of ET-1 or vehicle to the brain parenchyma after local craniotomy was performed as described [43]. Under isoflurane anesthesia, the animal head was placed in the stereotactic apparatus. A midline incision on the skin overlying the calvarium between the most caudal aspects of the eye to the ears was made. Bregma was determined and used for orientation, so that the drilling points for ET-1 injection side could be calculated (anterior–posterior: 0.0; medial–lateral: x − 4.2 and x − 3.2 according to Bregma) and marked. ET-1 (64 µmol in 5 µl PBS) was applied to the brain surface by pipette. The bur hole was covered with a glass slip and the incision closed.

Prior to either surgery, mice were treated with butorphanol for analgesia. All mice were allowed to recover under a heating lamp. Mice were treated with Tramadol for analgesia, provided with stereofundin supplemented with 5% Glucose (1.5 mL, sc) and cages were kept on a heating mat for post-operative care.

### Microglia suppression

An additional group of mice (*n* = 20) underwent continuous microglial suppression by colony-stimulating factor-1 receptor inhibitor PLX5622 (MedChem Express) treatment [4]. Mice were fed a modified diet containing PLX5622 (1200 ppm, Research Diets, *n* = 10) or a matched control diet (*n* = 10) beginning 7 days prior to surgical stroke. Dietary CSF-1R inhibition was maintained for the full duration of the study. Animals underwent serial PET and MRI imaging as described.

### Radiochemistry

The TSPO-targeted radiotracer ^18^F-GE180 was synthesized using a semi-automated module, with high radiochemical purity, yield, and specific activity (450–600 GBq/µmol), as previously described [40].

### Whole-body small-animal PET

Serial ^18^F-GE180 PET images were acquired using a Siemens Inveon DPET (Knoxville, Tennesse) as previously described [7]. Briefly, mice were positioned prone in the scanning bed with the whole body centered in the field of view. ^18^F-GE180 (12.51 ± 1.29 MBq) was administered as a 150 µL bolus via a catheter inserted in the lateral tail vein. A dynamic 60 min image was acquired in list mode. A low-dose computed tomography (CT) scan was conducted afterward for anatomical coregistration. Images were histogrammed to 32 frames and reconstructed using an iterative algorithm. Details are provided in the online supplement.

### Cardiac and brain MRI

MRI was acquired to assess cardiac function and stroke size early (2-4d) and late (20d) after induction of stroke. Images were acquired using a Bruker 7 T small-animal MR scanner (PharmaScan 70/16). Cardiac imaging was performed as previously described [16]. Brain imaging used a mouse brain receive-only coil array (11,765 C3) in combination with a quadrature MRI transmit-only coil with active decoupling T11070. Mice were placed prone into the scanner and a T2-weighted 2D multi-slice multi*echo (MSME) sequence was acquired. A 3D modified driven equilibrium Fourier transform method (MDEFT) was applied to the image.

### Image analysis

The Inveon Research Workplace software (Siemens) was used for analysis of cardiac PET images. Regions of Interest (ROI) were defined for the heart by interactive thresholding, and tracer uptake was semi-quantitatively analyzed as injected dose per gram of tissue (%ID/g). ROIs were analyzed slice-by-slice to exclude voxels with overlap from liver or lung tracer activity to minimize spill-in. Tracer uptake in brain was analyzed using PMOD 3.7 Software (PMOD Technologies Ltd., Zurich, Switzerland). Briefly, PET images were coregistred to a batched MRI template, and brain atlas (mouse Mirrione T2) derived ROIs were applied [30]. The uptake (%ID/g) was calculated for at 40–60 min after tracer injection. Additionally, ROIs for comparison of the uptake in the stroke area were drawn and used for comparison of the ipsilateral versus the contralateral brain hemisphere. For more detailed information on ^18^F-GE180 uptake in the stroke area versus the healthy contralateral side and the rest of the brain, the hottest 10% of pixels of the stroke region ROIs were calculated and used as %ID/g max. For analysis of T2-weighted brain MR, images were imported into ITK-Snap 3.8.0 [45], and contours were manually drawn around the hyperintense signal in sequential transaxial slices, which were then interpolated to calculate stroke volume (mm^3^). Cardiac function was analyzed using Segment v2.2 (Medviso). Endocardial and epicardial contours were traced on sequential short axis slices and interpolated to calculate ventricle geometry and volume over the cardiac cycle. Ejection fraction was calculated as LVEF = (LVVED-LVVES)/LVVED)*100, where LVV is the left-ventricular endocardial volume at end diastole (ED) or end systole (ES).

### Ex vivo workup

To confirm in vivo PET and MRI findings, mice were sacrificed for ex vivo validation at the terminal endpoint (22 days). Additional animals were sacrificed at the intermediate timepoints. Hearts and brains were rapidly excised, snap-frozen in liquid nitrogen-cooled Tissue Tec or isopentane, respectively. Tissue was stored at − 80 °C until analysis.

### In vitro autoradiography

For in vitro autoradiography, slides were incubated with ^18^F-GE180 (4 MBq/200 ml PBS) for 30 min. A washing step using PBS and water followed and finally slides were exposed to a high-resolution imaging plate (PerkinElmer) for 30 min in a light-impermeable cassette. For quantification, a standard curve of known concentration of 500–0 kBq was included in exposure in parallel. After exposure, images were digitalized using a Cyclon scanner (PerkinElmer). Digital images were analyzed in PMOD 3.7 software using an ROI encompassing the whole myocardium, and an ROI with differentiation between the ipsi- and contralateral cortex, hippocampus, and thalamus. The image densitometry was converted to a quantitative scale using the concurrently exposed standard curve of known concentration to determine a Bq/mm^3^ measurement which were regionally normalized to a control region to facilitate comparison across phosphor screens and tracer productions.

### Histology

Brains were stained with 2,3,5-triphenyltetrazolium chloride at acute timepoints to assess stroke region according to the manufacturer protocol [43]. Heart morphology was determined with hematoxylin/eosin (Sigma-Aldrich) and brain morphology was assessed by cresyl violet staining (Sigma-Aldrich) following the manufacturer protocols.

### Immunohistochemistry

Cryosections of heart (6 µm) and brain (14 µm) were stained for macrophages and microglia using for biotin-conjugated anti-CD68 (rat anti-mouse CD68, clone FA-11, Bio-Rad catalog number MCA1957B, 1:100) as previously described [40]. To localize TSPO, sections were stained with unconjugated rabbit anti-mouse TSPO primary antibody (rabbit anti-mouse TSPO, clone BAA04749, Novus Biologicals catalog no. NBP1− 45,769, 1:250) and biotin-conjugated goat anti-rabbit IgG secondary antibody (Novus Biologicals catalog no. NB7158, 1:1000). After quenching with 0.3% hydrogen peroxide, sections were incubated with streptavidin-peroxidase (1:100, Dako) for 30 min and were visualized with diaminobenzidine (Sigma-Aldrich) according to the manufacturer protocol. Non-specific signal as assessed without application of primary antibody. Sections were cleared in xylol and coverslipped with Eukitt mounting medium (Sigma-Aldrich).

### Immunofluorescence

Immunofluorescence staining identified colocalization of TSPO and CD68. Sections fixed in acetone for 20 min, then washed in PBS, and blocked for 1 h in 10% horse serum. After rinsing with PBS, sections were stained with primary antibody against CD68 (AlexaFluor 488-conjugated rat anti-mouse CD68, clone FA-11, Bio-Rad catalog no. MCA1957A488, 1:100) and TSPO (unconjugated rabbit anti-mouse TSPO, clone BAA04749, Novus Biologicals catalog no. NBP1 − 45,769, 1:250) for 1 h. Sections were then washed and incubated for with secondary antibody (AlexaFluor594-conjugated donkey anti-rabbit IgG, BioLegend catalog no. 406418, 1:1000). Following a final wash in PBS and nuclear staining using 4’,6-Diamidin-2-phenylindol (DAPI), sections were cleared in xylol and cover slipped using fluorescence mounting medium (Dianova).

### Fluorescence-associated cell sorting

To determine CD45 cell content in the left ventricle, a subgroup of mice was sacrificed 7 days after MCAo or sham surgery under isoflurane anesthesia. Cell sorting and flow cytometry for CD45^+^ cardiac cells were performed as described [16, 39]. Briefly, hearts were harvested, rinsed, and atria and right ventricle removed. The weighted left ventricle was minced under lysis buffer [PBS containing 1 mg/ml Collagenase D, 100U/ml DNase I, 2.4 mg/ml Dispase, 3% fetal calf serum (FCS)] and placed in a MACS C-tube for gentle cell dissociation (Miltenyi Biotec, Germany). Samples were incubated at 37 °C dissociated with periodic rotation according to the manufacturer’s protocol. The resulting cell suspension was filtered into 50 ml Falcon tubes using a 40 µm cell strainer, and centrifuged at 300 g for 7 min at 4 °C. The pellet was resuspended in 2 ml FACS buffer (in PBS: 4%FCS, 2 mM EDTA) and transferred to a 2 ml tube. Cells were then centrifuged at 300 g for 5 min at 4 °C, and the resulting pellet resuspended in 200 µl of FACS buffer containing CD16CD32 antibody (clone 2.4G2, mouse BD Fc Block, BD Biosciences catalog no. 553142, dilution 1:100) for 10 min at 4 °C. Cells were then stained using CD45-Brilliant Violet 570 (clone 30-F11, catalog no. 103135; dilution 1:250), and samples were incubated at 4 °C in the dark for 20 min. Cells were rinsed with 1 ml of FACS buffer, and centrifuged 300 g*,* 5 min at 4 °C, and the final pellet was resuspended in 300 µl of FACS buffer and filtered into a clean test tube. CD45 + cells were collected and counted using an FACSAria IIu instrument (Becton Dickinson, NJ, USA).

### Statistics

All data are presented as mean ± standard deviation. Statistical analysis was performed using GraphPad Prism (GraphPad Software La Jolla, CA, USA, Version 6.01). No Each symbol represents an individual animal. Multiple hypotheses are tested in this study. No experiment-wide or across-test multiple test correction has been applied. For comparisons of multiple groups, one-way ANOVA with Sidak's post hoc test was used. For assessing the difference in TSPO signal over time in individual groups, one-way ANOVA with Tukey’s post hoc test was used. For comparison of two groups, unpaired Student’s *t* test was used. Repeated measures in individual animals were compared using paired Student’s *t* test. Pearson product–moment correlation coefficient evaluated linear regression between continuous variables. For statistical parametric mapping of restricted voxels, Spearman correlation was used. Statistical significance was considered at *p* < 0.05.

## Results

### Transient MCA occlusion evokes extensive cerebral ischemic injury with persistent neuroinflammation over 21d

**Fig. 1:**
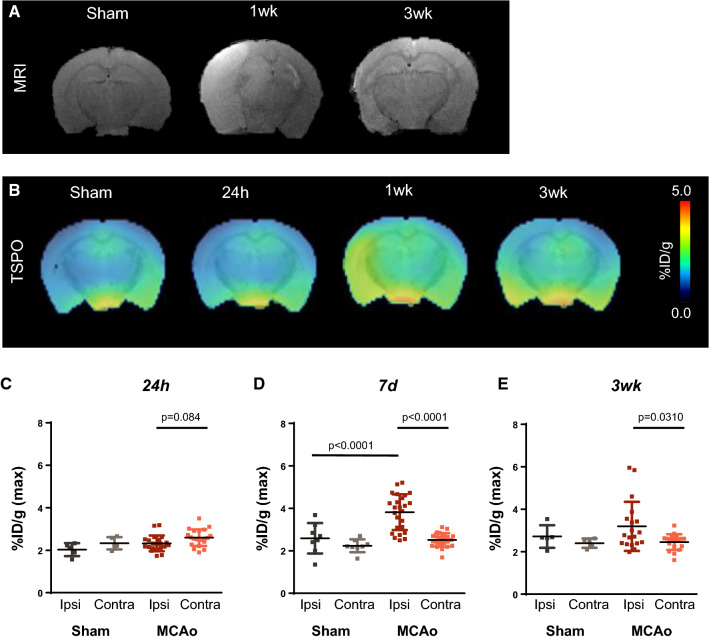
Serial multimodality imaging of infarct region and inflammation after middle cerebral artery occlusion (MCAo). **A** Representative T2-weighted MRI images identify stroke core and penumbra at 1 and 3 weeks after surgery. **B** Serial averaged PET images of translocator protein (TSPO) display localized neuroinflammation in the operated ipsilateral hemisphere (left) beginning from 7 days after MCAo. Semi-quantitative analysis calculates the average % injected dose (ID)/g in the highest 10% of voxels in the ipsilateral (ipsi) or contralateral (contra) hemisphere at **C** 24 h, **D** 7 days and **E** 3 weeks after MCAo or sham surgery (sham 24 h *n* = 5, 7 days *n* = 8, 3week *n* = 5; MCAo 24 h *n* = 23, 7 days *n* = 26,3week *n* = 17); one-way ANOVA, Sidak post hoc test

Occlusion of the left MCA for 30 min followed by reperfusion generated a prominent stroke in the left hemisphere of the murine brain. Upon recovery, animals exhibited transient behavioral abnormalities consistent with stroke including unidirectional circling and impaired righting reflex. T2-weighted MRI 2d after injury revealed a hyperintense signal of variable size in the injured ipsilateral hemisphere (Fig. 1A). The absolute stroke size differed among individuals, with a median extent of 44.7mm^3^ (range: 5.1–139.6mm^3^) (Suppl. Figure 1A). To assess stroke-induced neuroinflammation, we performed serial PET imaging with ^18^F-GE180 and evaluated TSPO upregulation in the MRI-defined stroke territory. Averaged brain images demonstrated elevated TSPO signal emanating from the ipsilateral hemisphere (Fig. 1B) over the time course of 21d. At 24 h after surgery, no difference in signal was detected between the ipsilateral and contralateral hemispheres (2.3 ± 0.4 vs 2.6 ± 0.4% injected dose (ID)/g, *p* = 0.084), nor between MCAo and sham-operated mice (2.3 ± 0.4 vs 2.0 ± 0.3%ID/g, *p* = 0.388, Fig. 1C). Conversely at 7 days, the TSPO signal was elevated by 52% in the injured ipsilateral hemisphere compared to the contralateral (3.8 ± 0.8 vs 2.5 ± 0.3%ID/g, *p* < 0.001, Fig. 1D), and by 47% compared to sham (3.8 ± 0.8 vs 2.6 ± 0.7, *p* < 0.001). This difference declined by 21 days (Suppl Fig. 1B), but remained 30% higher in the ipsilateral compared to contralateral hemisphere after MCAo (3.2 ± 1.2 vs 2.6 ± 0.4%ID/g, *p* = 0.031, Fig. 1E).

### Topical application of ET-1 to brain cortex after craniotomy generates less severe ischemic injury

**Fig. 2:**
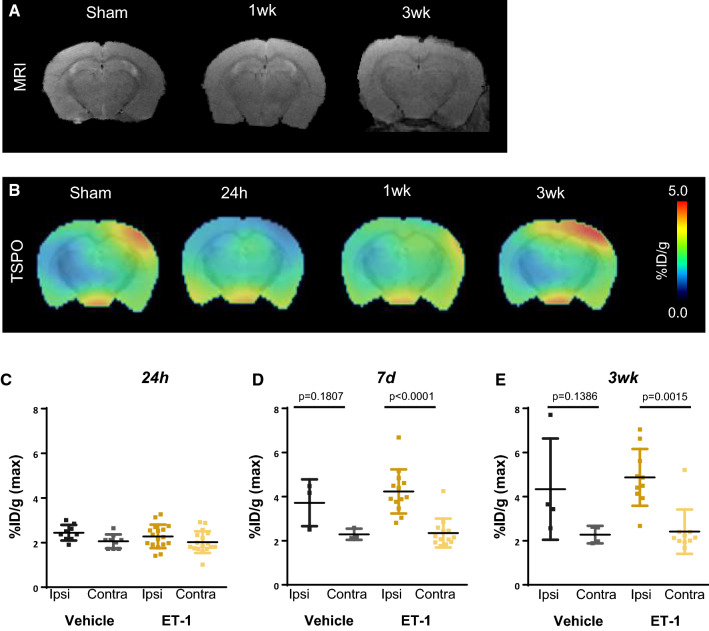
Serial multimodality imaging of brain morphology and inflammation after craniotomy and topical endothelin-1 (ET-1) application. **A** Representative T2-weighted MRI images cannot distinguish a stroke core penumbra region at 1 or 3 weeks after surgery. **B** Serial averaged TPSO PET images display gradually increased neuroinflammation in the operated ipsilateral hemisphere (right) beginning from 7 days and increasing to 21 days after craniotomy. Semi-quantitative analysis reveals elevated %ID/g in the hottest 10% of voxels in the ipsilateral (ipsi) or contralateral (contra) hemisphere at **C** 24 h, **D** 7 days, and **E** 3 weeks after craniotomy and vehicle or ET-1 application (vehicle 24 h *n* = 6, 7 days *n* = 3, 3 week *n* = 04; ET1 24 h *n* = 14, 7 days *n* = 13, 3week *n* = 10); one-way ANOVA, Sidak post hoc test

To obtain a regional stroke of smaller size, we performed a localized craniotomy and topical application of the vasoconstrictor ET-1 to the right hemisphere brain parenchyma. On recovery, animals exhibited minor behavioral signs of stroke, but tended to recover faster than from MCAo surgery without prolonged behavioral changes. T2-weighted MRI did not identify signal hyperintensity at the site of craniotomy and ET-1 application (Fig. 2A), reminiscent of transient ischemia rather than overt stroke. Serial PET imaging with ^18^F-GE180 revealed gradually increasing TSPO signal from the injured right hemisphere cerebral cortex at the location of craniotomy, but did not distinguish between vehicle and ET-1 application (Fig. 2B). At 24 h, ipsilateral and contralateral hemispheres showed similar basal levels of TSPO signal and no difference was observed at the site of ET-1 or vehicle application (2.3 ± 0.5 vs 2.4 ± 0.3%ID/g, *p* = 0.824, Fig. 2C). By 7 days after surgery ET-1 animals exhibited an 80% increase in TSPO signal at the site of craniotomy compared to the contralateral side (4.2 ± 1.0 vs 2.3 ± 0.7%ID/g, *p* < 0.001, Fig. 2D), but this elevation was similar in vehicle-treated mice (4.2 ± 1.0 vs 3.7 ± 1.1%ID/g, *p* = 0.773). This unilateral elevation in TSPO signal in ET-1 and vehicle craniotomy animals remained at 21 days (Fig. 2E), with no change in the contralateral hemisphere over time (Suppl Fig. 2). This similar TSPO upregulation between ET-1 and vehicle application suggested an inflammatory response to epithelium from the craniotomy alone.

### MCAo but not ET-1 stroke induces unilateral neuroinflammation on ex vivo tissue analysis

**Fig. 3:**
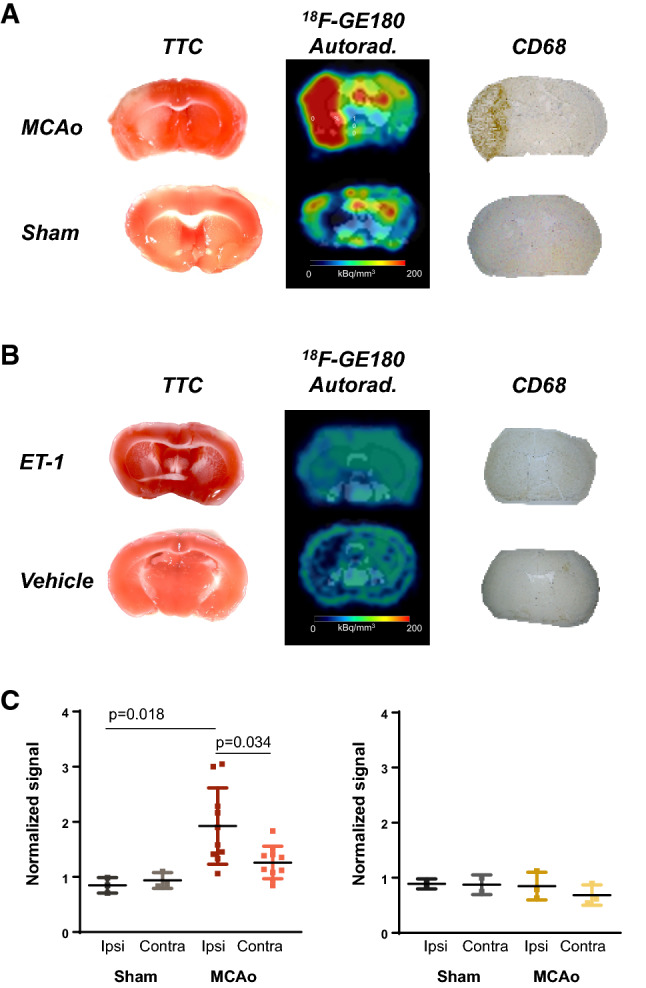
Ex vivo autoradiography and histology confirm localized neuroinflammation in MCAo but not ET-1 model. **A** Representative triphenyltetrazolium chloride (TTC) staining at 24 h after MCAo or sham surgery identifies the stroke area (red = viable tissue, white = infarct area) in the ipsilateral (left) hemisphere. At 7 days after injury, the stroke region corresponds to elevated TSPO-targeted ^18^F-GE180 autoradiography signal and CD68-positive macrophages (brown). **B** Co-Immunofluorescence with TSPO and CD68 depicts correspondence of TSPO and CD68 cells. **C** Representative TTC staining at 24 h denotes limited stroke damage to the ipsilateral (right) hemisphere after ET-1 topical application. ^18^F-GE180 autoradiography and CD68 immunostaining describe limited neuroinflammation at the stroke site 7 days after injury. Representative sections selected from the average tracer uptake in vivo. **D** Semi-quantitative autoradiography signal in MCAo and ET-1 stroke mice at 7 days after injury, normalized to sham contralateral hemisphere

To verify that in vivo images represented local neuroinflammation and to circumvent potentially reduced tracer delivery due to reduced blood flow in the ischemic region, brain sections were obtained from stroke and control mice, incubated with ^18^F-GE180 for 30 min, and exposed to phosphor screens. Consistent with the in vivo images, autoradiography revealed imbalanced tracer distribution in MCAo brains at 7 days after injury, localized to the stroke region defined by TTC staining (Fig. 3A). No comparable difference in signal was identified in sham-operated animals. Neither ET-1 nor vehicle cortical application after craniotomy displayed differences in activity accumulation between the ipsilateral and contralateral hemispheres (Fig. 3B), supporting the notion of epithelial inflammatory response to craniotomy. Immunostaining in adjacent brain sections demonstrated localized increase in CD68 + microglia in the stroke region after MCAo, corresponding to the elevated autoradiography signal (Fig. 3A). Microglia were not clearly visualized at 7 days after craniotomy and ET-1 or vehicle application to the cortical parenchyma (Fig. 3B). Semi-quantitative analysis of autoradiography sections at 7 days after surgery, normalized to the contralateral sham region signal confirmed upregulation of TSPO signal in the injured cortex in the MCAo model that was absent in the ET-1 model (Fig. 3C). Notably, the degree of increase was comparable to the in vivo PET signal. Moreover, regional analysis of in vitro autoradiography at 7 days after MCAo or sham surgery identified localized increase in TSPO signal in the ipsilateral cortex, hippocampus, and thalamus relative to the contralateral structures at 7 days after injury (Suppl Fig. 3), consistent with the brain region supplied by the MCA.

### MCAo stroke induces persistent modest cardiac dysfunction

**Fig. 4:**
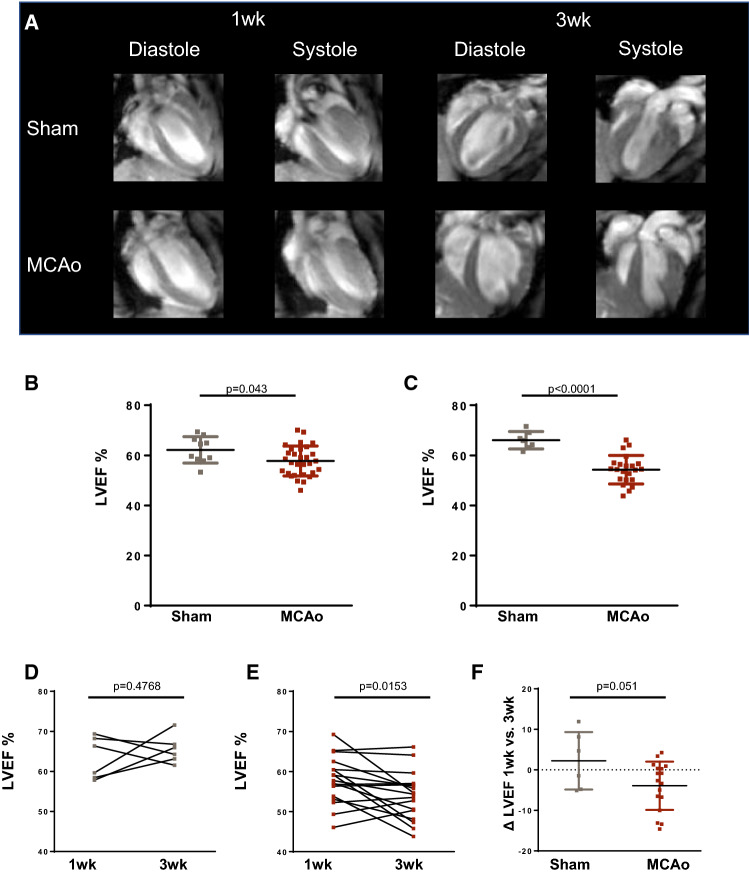
Serial assessment of cardiac function after MCAo stroke. **A** Representative cardiac MRI images at end systole and end diastole in sham and MCAo animals at 1–3 weeks after surgery. Quantitative assessment of left ventricle ejection fraction (LVEF) describes a modest reduction at **B** 1 week and **C** 3 weeks after surgery (sham 1 week *n* = 10, 3 week *n* = 7; MCAo 1 week *n* = 20, 3 week *n* = 18); Student’s unpaired *t* test. Evaluation of repeated LVEF measurements in **D** sham or **E** MCAo mice reveals a significant decline in cardiac function. **F** The average change in ejection fraction (Δ LVEF) from 1 to 3 weeks shows decline in function after MCAo compared to sham (sham *n* = 6; MCAo *n* = 17); Student’s paired *t* test

To determine the effects of cerebral stroke on cardiac function, ventricle volumes at end systole and end diastole were assessed from cardiac MR images at 1 week (2–6 days) and 3 weeks after MCAo or ET-1 stroke (Fig. 4A). At 1 week after MCAo, left ventricle ejection fraction was modestly reduced compared to sham animals (57.7 ± 6.0 vs 62.2 ± 5.2%, *p* = 0.043, Fig. 4B) and remained depressed at 3 weeks (54.3 ± 5.7 vs 66.1 ± 3.5%, *p* < 0.001), with a parallel decrease in stroke volume (Suppl. Figure 4). By contrast, in the absence of appreciable stroke in the ET-1 model, ejection fraction were unchanged at 1 or 3 weeks after surgery (Suppl Fig 5). Serial interrogation of individual animals revealed a moderate but persistent decline in ejection fraction between 1 and 3 weeks after MCAo-induced stroke (*p* = 0.015), whereas sham-operated animals showed maintained contractile function (Fig. 4D–F), suggesting persistent cardiac dysfunction in response to cerebral injury.

### MCAo stroke evokes increased TSPO expression in the heart at subacute and chronic timepoints after injury

**Fig. 5:**
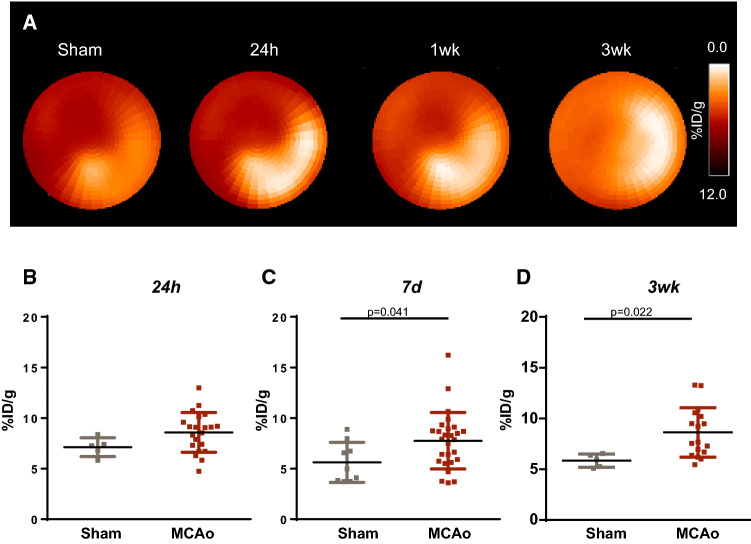
Molecular imaging of TSPO in the heart after MCAo or sham surgery. **A** Representative polar maps show global left ventricle TSPO PET signal after MCAo or sham surgery. Semi-quantitative analysis identifies **B** similar TSPO signal in the left ventricle at 24 h after surgery, and elevated signal at **C** 7 days and **D** 3 weeks after stroke compared to age-matched sham (sham 24 h *n* = 5, 7 days *n* = 9, 21 days *n* = 5; MCAo 24 h *n* = 21, 7 days *n* = 27, 3 week *n* = 17); Welch’s *t* test for unequal variance

**Fig. 6:**
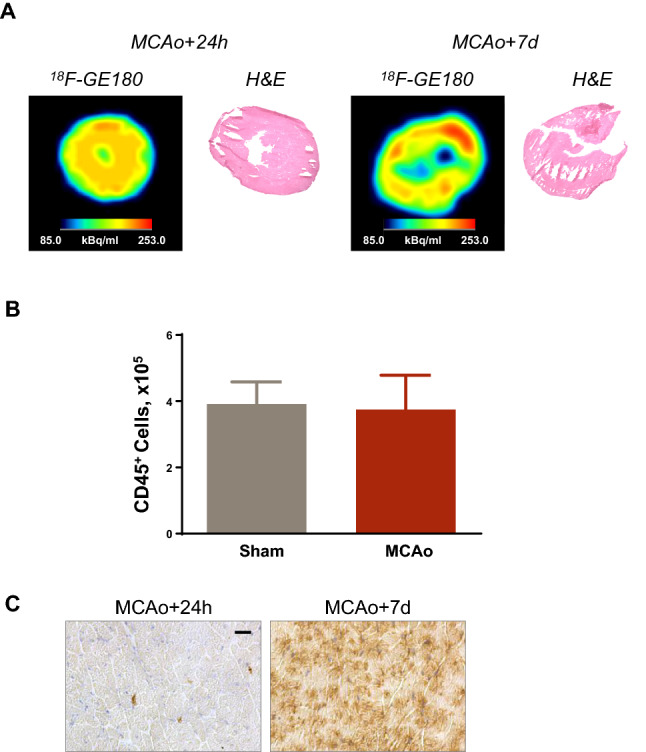
Ex vivo workup of heart confirms elevated TSPO without cardiac inflammation. **A** In vitro autoradiography displays uniform 18F_GE180 distribution in the left ventricle after MCAo with elevated signal intensity at 7 days relative to 24 h. **B** Fluorescence-associated cell sorting demonstrates similar CD45-positive leukocyte content in the left ventricle at 7 days after MCAo or sham surgery (sham *n* = ; MCAo *n* =). **C** Immunostaining identifies increased TSPO content in heart after MCAo at 7 days compared to 24 h, localized to cardiomyocytes. Representative images selected based on average of in vivo imaging quantitative values

Since inflammatory cells contribute to remodeling after cardiac injury and cerebral stroke evokes systemic inflammation, we assessed TSPO PET signal in the myocardium as a potential marker of leukocyte infiltration (Fig. 5A). At 24 h after MCAo surgery, a modest and diffuse trend to an increase in TSPO signal was observed in the global myocardium compared to sham (Fig. 5B). This difference was more prominent at 7 days (7.8 ± 2.8 vs 5.6 ± 2.0%ID/g, *p* = 0.041, Fig. 5C), and remained elevated at 21 days after injury (8.6 ± 2.4 vs 5.8 ± 0.7%ID/g, *p* = 0.022, Fig. 5D). TSPO signal was persistently elevated in the myocardium after MCAo, whereas sham animals showed a modestly higher signal in the acute stage, possibly related to systemic response to the surgery (Suppl Fig 6). Autoradiography confirmed enriched TSPO signal from the myocardium after MCAo relative to sham at 7 days (Fig. 6A). To evaluate the infiltration of inflammatory leukocytes to the myocardium, we performed FACS analysis of left ventricle at 7 days, which revealed comparable CD45 + leukocyte density in MCAo and sham hearts (Fig. 6B). Immunostaining showed limited CD68 cell content after MCAo compared to sham, but elevated TSPO staining predominantly localized within cardiomyocytes (Fig. 6C).

### Stroke size and neuroinflammation as a predictor of cardiac function

**Fig. 7:**
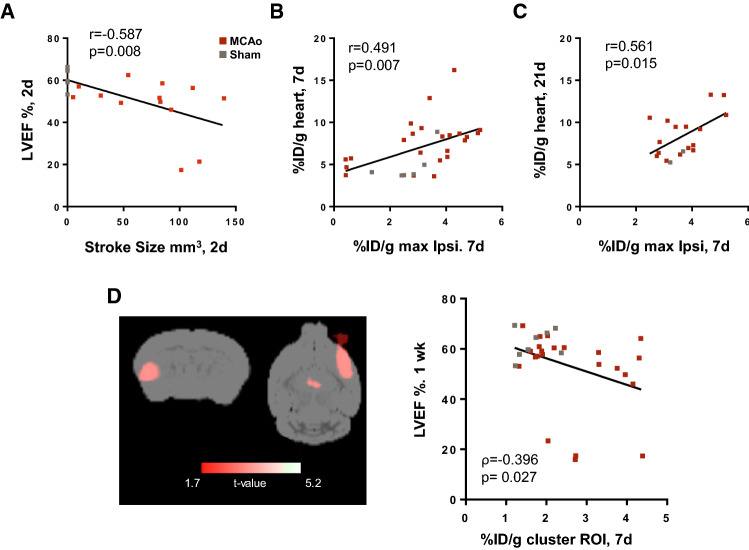
Stroke size and neuroinflammation intensity parallel cardiac function and TSPO signal. **A** Pearson correlation analysis demonstrates that early T2-MRI derived stroke size can predict subsequent ejection fraction at 21 days after MCAo (sham *n* = 6; MCAo *n* = 13). **B** Pearson correlation analysis describes the brain–heart axis in the correlation between stroke region and cardiac TSPO PET signal at **B** 7 days (sham *n* = 6, MCAo *n* = 23) and **C** 3 weeks after MCAo surgery (sham *n* = 2, MCAo *n* = 16). **D** Voxelwise statistical parametric mapping analysis identified a cluster of voxels in the injured hemisphere where TSPO signal was significantly associated with cardiac dysfunction. Spearman correlation between the TSPO signal at 7 days and cardiac function at 7 days after MCAo generated from SPM analysis (sham *n* = 8, MCAo *n* = 26)

Regression analysis of early T2-weighted MR-derived stroke size with contractile function demonstrated that larger stroke size was associated with worse cardiac function (Fig. 7A). Notably, TSPO signal in the ipsilateral hemisphere of MCAo mice also significantly correlated with myocardial ^18^F-GE180 uptake (Fig. 7B). Moreover, the TSPO signal in the ipsilateral hemisphere at 7 days after injury predicted higher TSPO signal in the LV by d21 (Fig. 7C), suggesting that microglial activation may contribute to contractile and mitochondrial dysfunction, even in the absence of overt inflammatory cell infiltration.

### Reduced stroke severity did not spare acute or chronic contractile function

Based on the relationship between stroke size and cardiac function, we investigated whether less severe stroke would spare contractile function by reducing the MCA occlusion time to 20 min. Reduced occlusion time resulted in consistent elevation of the TSPO PET signal compared to sham-operated animals, similar to the 30 min occlusion time (Suppl Fig 7). Contractile function was reduced at 1–3 weeks compared to sham but did not differ between 20 and 30 min occlusion time (Suppl Fig 7).

### Microglial suppression lowers acute and chronic TSPO signal and impacts chronic cardiac function

**Fig. 8:**
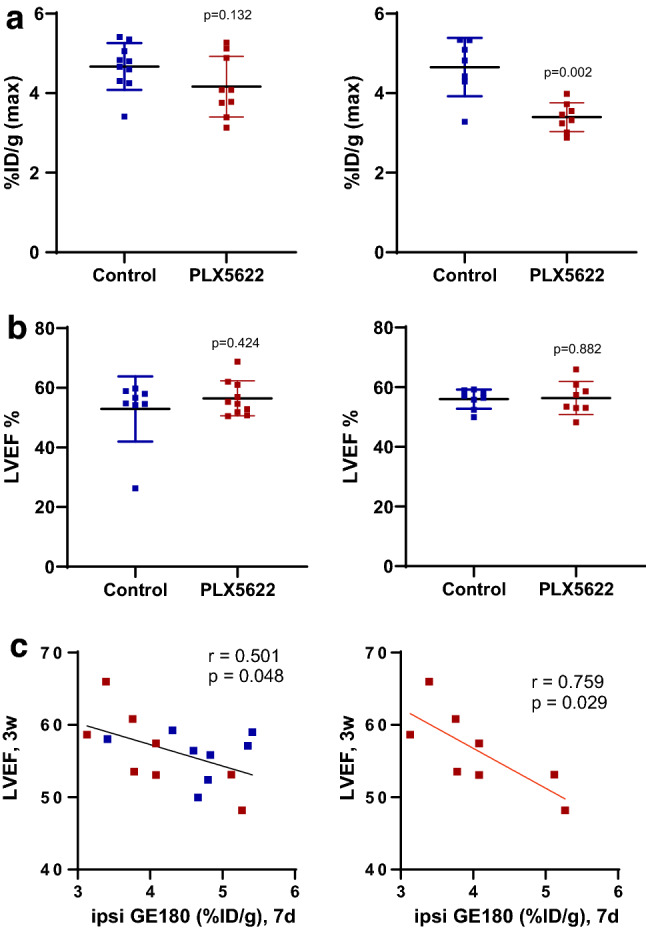
Effective microglia depletion lowers brain TSPO signal and marginally spares cardiac function. **a** Semi-quantitative uptake of ^18^F-GE180 in the ipsilateral cortex at 7 days (left) and 21 days (right) after MCAo with continuous suppression of microglia by CSF-1R inhibitor PLX5622 compared to control diet. **b** Left ventricle ejection fraction (LVEF) at 1 week (left) and 3 weeks (right) after MCAo surgery displays no direct impact of PLX5622 on cardiac function. **c** Pearson analysis describes an inverse correlation between 7 day TSPO signal in injured ipsilateral hemisphere and 3 weeks LVEF including PLX5622 and control diet MCAo mice (left) and PLX5622 treated alone (right), control diet *n* = 8–10 control, PLX5622 diet *n* = 8–9

Since microglial activation 7 days after injury particularly at the insular cortex was associated with worse contractile function, we suppressed microglia in a group of mice by chronic treatment with the CSF-1R inhibitor PLX5622 [4]. There was a trend to reduced TSPO signal at 7 days after MCAo in the injured hemisphere which was significantly reduced at 21 days after injury (Fig. 8A). By contrast, contractile function at 1–3 weeks after MCAo was similar between PLX5622 and control mice (Fig. 8B). However, regression analysis revealed that mice with lower TSPO signal at 7 days after injury tended to exhibit higher ejection fraction at 3 weeks (Fig. 8C), suggesting that more effective microglial suppression during stroke progression may partly spare cardiac function.

## Discussion

Beyond local ischemic damage and neurological morbidity, cerebral stroke is associated with cardiac dysfunction. Here, we employed two mouse models of focal cerebral stroke and multimodality molecular imaging to gain mechanistic insights into the relationship between neuroinflammation and adverse cardiac outcome following ischemic brain injury. We have demonstrated that the extent and severity of stroke directly correlates with early and progressive impairment of contractile function. Higher inflammatory cell content in the stroke core and penumbra within the first days of ischemic damage identified non-invasively by PET imaging of TSPO is associated with a parallel increase in cardiac TSPO signal and impaired contractile function. Notably, this cardiac TSPO signal is independent of inflammatory cell infiltration, supporting the notion that TSPO PET also identifies mitochondrial dysfunction in cardiomyocytes. These observations indicate potential value of multimodality molecular imaging including T2-MRI to identify the infarct core and penumbra, and whole-body TSPO imaging to identify risk of cardiac dysfunction after cerebral stroke.

Historical clinical data indicate that coronary heart disease is the second most common cause of mortality following cerebral stroke, responsible for 19% of total deaths [10, 25]. The 5-year risk for myocardial infarction and sudden cardiac death after stroke is 10.6%, roughly twice the expected rate in the healthy population [1, 10]. For transient ischemic attack, subsequent cardiac injury was more fatal than incident stroke [18]. Cardiac-specific injury commonly occurs after clinical stroke, wherein 29% of patients exhibit reduced ejection fraction within the first 3 months of injury [8, 34]. When the stroke-damaged region encompasses the right insular cortex, this incidence rises to 90% [3, 29]. Accordingly, clinical recommendations highlight the importance of assessing cardiovascular risk after primary stroke and identifying unrecognized coronary heart disease [1]. However, the precise mechanisms underlying the interaction between ischemic stroke and cardiac damage remain equivocal and are difficult to assess at the tissue level using the conventional blood biomarker measurements [17]. Multimodality imaging affords the opportunity to simultaneously define pathogenetic processes in the brain and heart.

In the present study, we characterized two mouse models of cerebral stroke using T2-weighted MRI to define the stroke focus and penumbra early after injury and TSPO-targeted PET to assess neuro- and cardiac inflammation. Intraluminal occlusion of the MCA is well established to generate a stroke of variable severity, depending on the duration of occlusion [38]. The severity of ischemic damage can vary between individual animals, generating a range of focus and penumbra extent that can be detected by T2-weighted MRI [28]. TTC staining revealed early ischemic injury in the ipsilateral hemisphere in parallel with early and sustained microglia content in the stroke core and penumbra regions. By contrast, the ET-1 stroke model generates mild stroke symptoms. We observed only mild indications of tissue damage by TTC staining and no behavioral abnormalities at acute or chronic timepoints. While some prior studies have demonstrated localized neuroinflammation and neurodegeneration at the site of cerebral ET-1 application [43], others suggest that the transient vasoconstrictive effect does not generate significant stroke damage in mice [19], possibly due to lack of penetration to deeper brain regions and the short biologic half-life of ET-1 [44]. Indeed, the short and localized action of ET-1 may more accurately model transient ischemic attack, which is less associated with cardiac dysfunction than overt stroke [1].

It should be noted that in the present study, practical surgical considerations meant that the ischemic hemisphere for MCAo and topical ET-1 were different. It is conceivable that asymmetry of the rodent brain evokes different effects of left- and right-sided stroke. Left hemisphere ischemia is thought to produce more severe sensorimotor impairment, whereas right hemisphere ischemia is more associated with cognitive impairment [46]. Some evidence suggests that the left insular cortex may be more strongly linked to cardiac dysfunction [22], though troponin T levels reflecting cardiac damage are similarly elevated with left- or right-sided stroke [3]. While we cannot exclude the possibility that the MCAo cardiac dysfunction was exacerbated by its location in the left hemisphere, limited histologic indication of stroke damage in the ET-1 model likely impacted the lack of cardiac damage.

In the present study, the extent of ischemic core and penumbra as well as edema identified by T2-weighted MRI early after MCAo surgery was associated with a modest and persistent decline in contractile function. Earlier studies have shown that stroke impairs acute and chronic cardiac function [3, 22]. Due to the common involvement of systemic inflammation in the progression of heart failure, we hypothesized that the immune system may be a crucial component of the brain–heart axis after ischemic stroke. Previous reports have identified systemic inflammation in response to focal cerebral ischemic injury after MCAo [21]. Indeed, peripheral leukocyte mobilization and infiltration of the central nervous system are thought to contribute to stroke expansion and worse cognitive outcomes [9, 42]. Circulating monocytes, macrophages, and dendritic cells are increased after acute stroke, which also infiltrate the ischemic territory through the compromised blood–brain barrier [37]. Within 24 h of stroke, pro-inflammatory cytokines are elevated at the site of ischemia, leading to migration of peripheral leukocytes, including neutrophil migration from bone marrow [9]. Accordingly, the extent of T2-derived edema correlated with the TSPO signal in the ipsilateral hemisphere after MCAo, and predicted persistent inflammation in the brain at 3 weeks. Interestingly, neuroinflammation was persistently present at 3 weeks after MCAo-induced stroke, indicating a sustained inflammatory response to the initial injury. Notably, strategies to reduce systemic inflammation including splenectomy to reduce myeloid cell mobilization and inhibition of pro-inflammatory cytokines interleukin-1β or tumor necrosis factor-α result in a smaller stroke size after MCAo in rodents [2, 23, 24]. Alternatively, suppression of polymorphonuclear neutrophils by therapeutic antibody lowers accumulation of CD45 leukocytes in the stroke region with a parallel increase in endothelial cell function and smaller infarct size [15]. Complete suppression of neutrophils can interfere with novel therapeutic strategies after MCAo stroke such as small extracellular vesicles [13], supporting the notion that timing of anti-inflammatory interventions may be critical. These observations suggest a systemic inflammatory response to MCAo which could influence cardiac function. However, despite increased TSPO PET signal in the heart after MCAo, FACS analysis demonstrated no definable increase of CD45 leukocyte density in the heart.

Notably, TSPO expression is not restricted to peripheral leukocytes, and is robustly expressed in mitochondria of cardiomyocytes [41], where it is involved in cholesterol transport, oxidative stress, and substrate utilization [20]. Indeed, TSPO is upregulated in chronic heart failure [7, 40, 41], and inhibition of TSPO is cardioprotective after myocardial ischemia [33]. The increase in cardiac TSPO signal after cerebral stroke may be indicative of mitochondrial dysfunction and increased exome cycling, as described recently in mouse models of heart failure [31]. Nonetheless, the TSPO signal in the heart correlated with the severity of contractile impairment, suggesting a mechanistic contribution of mitochondrial dysfunction to stroke-induced cardiac damage, independent of systemic inflammation and immune cell mobilization.

Surprisingly, induction of less severe stroke by shorter duration ischemia did not spare contractile function in mice after MCAo. Because the dysfunction is modest and varied between individual mice, significant improvement in ejection fraction is difficult to demonstrate. Microglial suppression by CSF-1R inhibitor treatment resulted in incomplete dampening of the cortex TSPO signal after MCAo, similar to prior reports [4]. The lack of significant reduction at 7 days likely reflects contribution of peripheral immune cells to the penumbra inflammation early after ischemic stroke. By contrast, significant reduction of TSPO signal at 21 days after injury suggests that microglia are the primary contributors to chronic neuroinflammation after MCAo. Despite ineffective sparing of cardiac function by CSF-1R inhibition, the inverse correlation of early injured hemisphere TSPO signal with late contractile function supports the notion that regional neuroinflammation affects cardiac function.

While the present results suggest that systemic inflammation may have only minor impact on cardiac dysfunction after cerebral stroke, acute neuroinflammation may contribute to cardiac damage through other pathways. Damage to the insular cortex is associated with increased left ventricle pressure and sympathetic neuronal outflow, as measured by cardiac norepinephrine concentration [29]. Some studies have indicated that microglial activation in the paraventricular nucleus of the hypothalamus contributes to hypertension, which can be attenuated by local suppression of microglia [36]. Catecholamine surge in response to sympathetic activation has been implicated as a major contributor to acute cardiac events, and may contribute to chronic ventricle remodeling [8]. Further research is clearly warranted to better understand the link between cerebral stroke and cardiac-related mortality.

The present work demonstrates the value of multimodality imaging to define changes in the brain–heart axis after cerebral ischemic stroke. Importantly, the techniques applied here in mouse models are compatible with application in human patients [12]. The clinical manifestation of cardiac dysfunction following cerebral stroke is well recognized, but the mechanisms underlying this interaction remain unclear. The indication that the severity of neuroinflammation relates to the degree of cardiac dysfunction supports alternate therapeutic strategies to improve cardiac outcomes after cerebral stroke. It may further provide insights into the optimal timing of therapies that depend on leukocyte kinetics [13].

In conclusion, the extent and severity of neuroinflammation early after cerebral ischemia influences subsequent cardiac function. Sustained neuroinflammation is further associated with decline in left ventricle contractile function, supporting the notion of inter-organ communication between brain and heart via the immune system. Multimodality imaging provides valuable insights into this brain–heart networking and may identify novel therapeutic targets to improve cardiac outcome after stroke.

## Supplementary Information

Below is the link to the electronic supplementary material.

Supplementary file1 (DOCX 1280 KB)

## Data Availability

The data that support the findings of this study are available from the corresponding author upon reasonable request.

## Code availability

N/A.
