# Supplementary material for: Molecular imaging of the brain–heart axis provides insights into cardiac dysfunction after cerebral ischemia
Source: Basic Res Cardiol. 2022 Oct 24;117(1):52. doi: 10.1007/s00395-022-00961-4 (PMC9592646; doi:10.1007/s00395-022-00961-4)
Supplement: Supplementary file 1 — Supplementary file1 (DOCX 1280 KB) [file 395_2022_961_MOESM1_ESM.docx]

**Supplemental Figures**

**Supp. Fig 1: Brain morphology and serial neuroinflammation imaging in middle cerebral artery occlusion (MCAo) stroke. (A)** Evaluation of hypointense area as defined by T2-weighted brain MRI at 2d and 6d after MCAo (wk1) and 22d after MCAo (wk3). Hypointense signal includes penumbra and edema and decreases by 3wks. Time course of TSPO signal in the ipsilateral hemisphere after (**B**) MCAo or (**C**) sham surgery, and in the contralateral hemisphere after (**D**) MCAo and (**E**) sham surgery. Average and standard deviation. Statistics: one way ANOVA with Tukeys post-hoc test.

**Supp. Fig 2: Serial neuroinflammation imaging in craniotomy and endothelin-1 (ET-1) topical application model.** Time course of TSPO signal in the ipsilateral hemisphere after craniotomy and topical application of (**B**) ET-1 or (**C**) vehicle, and in the contralateral hemisphere after (**D**) ET-1 or (**E**) vehicle. Average and standard deviation. Statistics: one way ANOVA with Tukeys post-hoc test.

**Suppl. Figure 3: Semi-quantitative regional analysis of brain i*n vitro* autoradiography at 7d after MCAo.** Autoradiographs were applied to a defined MRI brain atlas to assess regional distribution. To allow comparison across multiple experiments, signal intensities were normalized to average activity in the contralateral hemisphere of sham animals. Ipsilateral cortex, thalamus and hippocampus are affected from MCAo. Statistics: one way ANOVA with Sidak’s post-hoc test.

**Suppl. Fig 4.: Left ventricle geometry and function after MCAo.** Serial quantitative assessment of ventricle volumes by cardiac MRI at (**A**) 1 week and (**B**) 3 weeks after MCAo or sham surgery. (**C**) Stroke volume was significantly reduced at 1 and 3 weeks after MCAo compared to sham. Statistics: Welch t-test for unequal variance.

**Suppl. Fig. 5: Serial evaluation of left ventricle function after craniotomy and topical ET-1 application**: **(A)** Representative cardiac MRI images at end systole and end diastole in ET-1 and vehicle animals at 1 and 3 weeks after surgery. Quantitative assessment of left ventricle ejection fraction (LVEF) describes similar function (**B**) 1 week and (**C**) 3 weeks after surgery. Statistics: Student’s unpaired t-test. Evaluation of repeated LVEF measurements in craniotomy plus (**D**) vehicle or (**E**) ET-1 reveals no significant change in LVEF over time. Statistics: Student’s paired t-test. (**F**) The average change in ejection fraction (Δ LVEF) from 1 to 3 weeks shows no difference compared to vehicle.

**Suppl. Fig. 6: Serial TSPO PET signal after MCAo or sham surgery.** (**A**) After MCAo TSPO signal in the left ventricle remains consistent from 24h to 3 weeks after surgery. (**B**) A modestly higher left ventricle TSPO signal is observed 24h after sham surgery that declines at 7d and 3 weeks after injury. This early signal elevation likely reflects a systemic response to the acute surgical intervention.


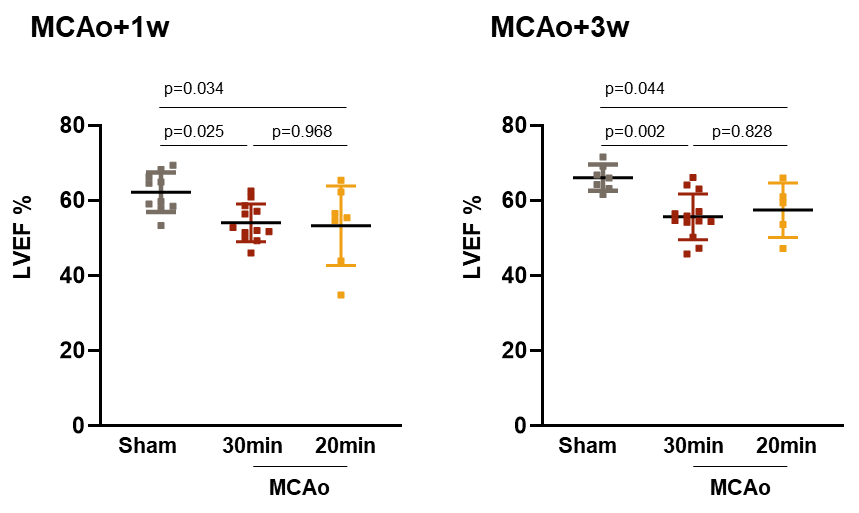


**Suppl. Fig. 7: Impact of shortened duration ischemia on cardiac function after MCAo.** Reduction of transient ischemia to 20min did not spare cardiac function relative to 30min of ischemia at either 1 week or 3 weeks after surgical stroke.
